# Long-term colonization exceeding six years from early infancy of *Bifidobacterium longum* subsp. *longum* in human gut

**DOI:** 10.1186/s12866-018-1358-6

**Published:** 2018-12-12

**Authors:** Kaihei Oki, Takuya Akiyama, Kazunori Matsuda, Agata Gawad, Hiroshi Makino, Eiji Ishikawa, Kenji Oishi, Akira Kushiro, Junji Fujimoto

**Affiliations:** 1 Yakult Honsha European Research Center for Microbiology ESV, Technologiepark 4, Gent-Zwijnaarde, Belgium; 2 Yakult Central Institute, 5-11 Izumi, Kunitachi, Tokyo, 186-8650 Japan

**Keywords:** Human gut microbiota, *Bifidobacterium longum* subsp. *longum*, Bacterial colonization, Long-term colonization, Infant, Child, Perinatal mother, Multilocus sequence typing (MLST), Culturing, Quantitative PCR

## Abstract

**Background:**

The importance of the gut microbiota at the early stage of life and their longitudinal effect on host health have recently been well investigated. In particular, *Bifidobacterium longum* subsp. *longum*, a common component of infant gut microbiota, appears in the gut shortly after birth and can be detected there throughout an individual’s lifespan. However, it remains unclear whether this species colonizes in the gut over the long term from early infancy. Here, we investigated the long-term colonization of *B*. *longum* subsp. *longum* by comparing the genotypes of isolates obtained at different time points from individual subjects. Strains were isolated over time from the feces of 12 subjects followed from early infancy (the first six months of life) up to childhood (approximately six years of age). We also considered whether the strains were transmitted from their mothers’ perinatal samples (prenatal feces and postnatal breast milk).

**Results:**

Intra-species diversity of *B. longum* subsp. *longum* was observed in some subjects’ fecal samples collected in early infancy and childhood, as well as in the prenatal fecal samples of their mothers. Among the highlighted strains, several were confirmed to colonize and persist in single individuals from as early as 90 days of age for more than six years; these were classified as long-term colonizers. One of the long-term colonizers was also detected from the corresponding mother’s postnatal breast milk. Quantitative polymerase chain reaction data suggested that these long-term colonizers persisted in the subjects’ gut despite the existence of the other predominant species of *Bifidobacterium*.

**Conclusions:**

Our results showed that several strains belonging to *B. longum* subsp. *longum* colonized in the human gut from early infancy through more than six years, confirming the existence of long-term colonizers from this period. Moreover, the results suggested that these strains persisted in the subjects’ gut while co-existing with the other predominant bifidobacterial species. Our findings also suggested the importance of microbial-strain colonization in early infancy relative to their succession and showed the possibility that probiotics targeting infants might have longitudinal effects.

**Trial Registration:**

TRN: ISRCTN25216339. Date of registration: 11/03/2016. Prospectively registered.

**Electronic supplementary material:**

The online version of this article (10.1186/s12866-018-1358-6) contains supplementary material, which is available to authorized users.

## Background

Several-hundred species of bacteria reside in the human gut [1] and a vast amount of evidence indicating the considerable influence of human gut microbiota on the health of the host has been accumulated [2]. In addition, functional differences of bacterial strains regarding host health have also been reported, such as their virulence [3], protective effects against pathogens [4], and immunoregulatory properties [5].

Currently, the importance of the gut microbiota at the early stage of life has been well investigated. Moreover, it is suggested that the gut microbial composition during this period is associated with the risk of diseases (e.g. allergy, asthma, and obesity) in the following life stages [6–8]. The human gut microbiota develops just after delivery and the composition dynamically shifts throughout the lifetime of the host [9]. The composition of an infant’s gut microbiota is influenced by various factors, such as the mode of delivery, diet, antibiotic usage during infancy, and host genetics [10]. In addition, Sharon et al. showed using a metagenomics approach that the shift of gut microbial composition occurs not only at the species level, but also at the strain level [11]. They also showed that some bacterial strains belonging to *Staphylococcus epidermidis* and *Propionibacterium* spp. sustainably colonize in the infant gut from 15 up to 24 days of age. The sustainable existence of the same bacterial strain in early infancy for a certain period of time has also been confirmed for *Clostridium difficile* [12] and several species of the genus *Bifidobacterium* [13, 14]. However, it remains unclear whether a bacterial strain colonizing in the human gut during early infancy represents just a temporal resident for a limited period or persists to colonize the gut in the following life stage(s).

*Bifidobacterium longum* subsp. *longum* is a unique bifidobacterial species in the human gut detected at high prevalence and abundance, not only from infants, but also from adults and seniors [13, 15–17]. Several studies have shown that some strains of this species afford health-promoting potential to their host [4, 18, 19]. Regarding their potential for continuous existence in the human gut, Shkoporov et al. reported the existence of two lineages of long-term colonizers that persist to colonize in the same subjects from 8 to 16 months of age through 6–10 years [20, 21]. Considering the dynamic shift of gut bacterial strains in early infancy [10], there is little doubt that considerable selective stress exists during this period. Therefore, questions still remain regarding whether a strain belonging to *B. longum* subsp. *longum* colonizing in the human gut in early infancy may have the potential to constitute a long-term colonizer by overcoming the selective stresses.

To investigate this question, we conducted a follow-up study focusing on a Belgian cohort in which we have previously confirmed that a number of *B. longum* subsp. *longum* strains were transmitted from the mother’s gut to that of the infant [13, 22] and were also shared between the infant’s gut and the mother’s postnatal breast milk [23]. In the present study, we confirmed the strain identity of *B. longum* subsp. *longum* isolates obtained from the fecal samples of an individual subject collected in both early infancy (in this study, the first six months of life) and childhood (approximately at six years of age). Furthermore, the analysis was expanded to the isolates obtained from their mothers’ perinatal samples (prenatal fecal and postnatal breast milk samples) to investigate whether the mother-infant transmitted strains were able to become long-term colonizers.

## Results

### *B. longum* subsp*. longum* strains focused on this study

**Fig. 1:**
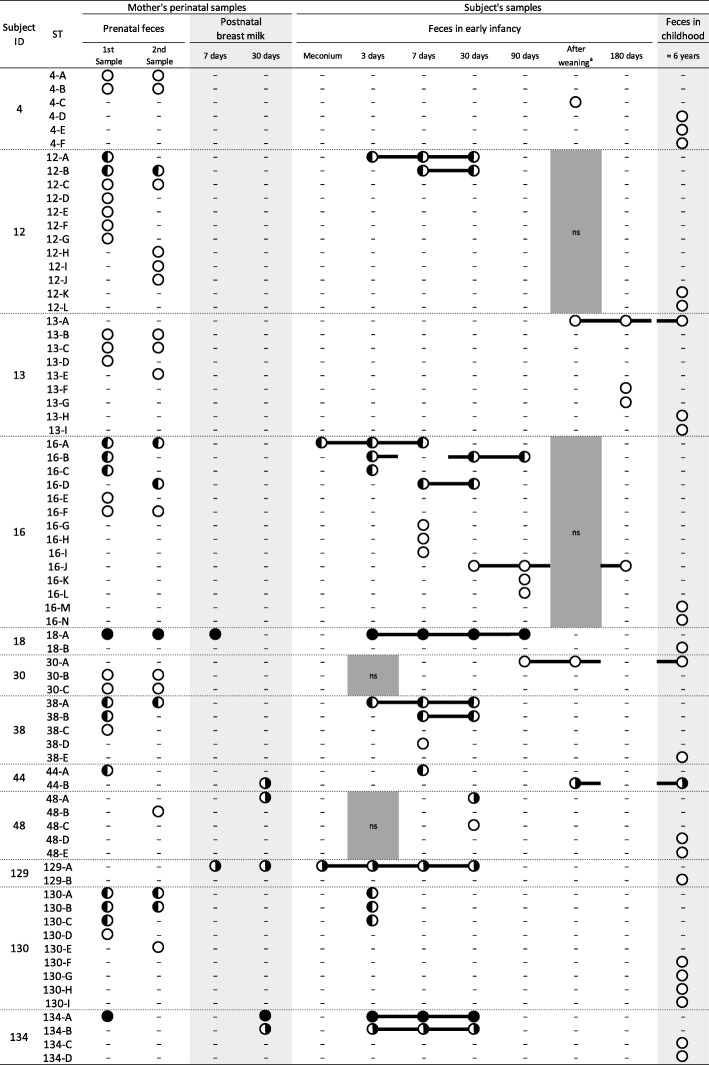
Detection points of the strains in the samples collected from 12 subjects and their mothers. Circles indicate detection of the strains. Circle color represents the detection type of the monophyletic strain as follows: black, detected from both the subjects’ fecal sample(s) in early infancy and their mothers’ perinatal sample(s); half black (left), detected from both the subjects’ fecal sample(s) in early infancy and their mothers’ prenatal fecal sample(s); half black (right), detected from both the subjects’ fecal sample(s) in early infancy and their mothers’ postnatal breast milk sample(s); white, detected from only the subjects’ fecal sample(s). A minus symbol means that no strain was detected. ns: no sample was collected. For each of the subject’s samples, strains that were confirmed to be monophyletic strains are linked with a line. ^*a*^ One week after the introduction of solids

For 12 out of the 49 subjects recruited for this follow-up study, *B. longum* subsp. *longum* isolates were obtained from the fecal samples collected in both early infancy and childhood (Additional file 1: Table S2). For these 12 subjects, in total, 462 isolates were obtained (Additional file 1: Table S2), which came from the fecal samples collected in early infancy (243 isolates) and in childhood (46 isolates), as well as from their mothers’ prenatal fecal (141 isolates) and postnatal breast milk samples (32 isolates). Based on the results from multilocus sequence typing (MLST) analysis, all isolates were classified into 140 strains belonging to 73 sequence types (STs) as shown in Fig. 1. Strains with certain STs were distinctively detected from a single subject’s fecal samples and the corresponding mother’s perinatal samples. More than one strain was detected from some subjects’ fecal samples collected in early infancy (max. Five strains/subject at a time point) and childhood (max. Four strains/subject at a time point), as well as some mothers’ prenatal fecal samples (max. Seven strains/subject at a time point) and the postnatal breast milk samples of the mother of subject 134 (two strains) (Table 1). Hereafter, strains originating from the same subjects’ fecal sample and the corresponding mothers’ perinatal samples sharing the same ST are defined as monophyletic strains.

**Table 1:** Count of monophyletic strains in the samples detected from 12 subjects and their mothers

Subject ID	Detection of the representative strains^a^											
	Mother’s perinatal samples				Subject’s samples							
	Prenatal feces		Postnatal breast milk		Feces in early infancy							Feces in childhood
	1st Sample	2nd Sample	7 days	30 days	Meconium	3 days	7 days	30 days	90 days	After weaning^b^	180 days	≈ 6 years
4	2	2	–	–	–	–	–	–	–	1	–	3
12	7	5	–	–	–	1	2	2	–	ns	–	2
13	3	3	–	–	–	–	–	–	–	1	3	3
16	5	3	–	–	1	3	5	3	4	ns	1	2
18	1	1	1	–	–	1	1	1	1	–	–	1
30	2	2	–	–	–	ns	–	–	1	1	–	1
38	3	1	–	–	–	1	3	2	–	–	–	1
44	1	–	–	1	–	–	1	–	–	1	–	1
48	–	1	–	1	–	ns	–	2	–	–	–	2
129	–	–	1	1	1	1	1	1	–	–	–	1
130	4	3	–	–	–	3	–	–	–	–	–	4
134	1	–	–	2	–	2	2	2	–	–	–	2

^a^ns, no sample was collected; −, no isolate was obtained

^b^One week after the introduction of solids

### Comparison of sequence type

A total of 14 monophyletic strains (ST 12-A, 12-B, 16-A, 16-B, 16-C, 16-D, 18-A, 38-A, 38-B, 44-A, 130-A, 130-B, 130-C, and 134-A) were detected from both the fecal samples of seven subjects in early infancy and the corresponding mothers’ prenatal fecal samples (Fig. 1). In comparison, six monophyletic strains (ST 18-A, 44-B, 48-A, 129-A, 134-A, and 134-B) were detected from both the fecal samples of five subjects in early infancy and the corresponding mothers’ postnatal breast milk samples.

From the same subjects’ fecal samples, three monophyletic strains (ST 13-A, 30-A, and 44-B) were detected in both early infancy and childhood and were classified as long-term colonizers (Fig. 1). These long-term colonizers were detected from 120, 90, and 110 days of age, respectively. A monophyletic strain of a long-term colonizer (ST 44-B) was also isolated from the mother’s postnatal breast milk sample collected at 30 days after delivery. No monophyletic strains obtained from a mother’s prenatal fecal sample were detected from the subject’s (offspring’s) fecal sample in childhood, although 14 such strains were detected from seven subjects’ fecal samples in early infancy.

### Comparison of allelic profiles

**Fig. 2:**
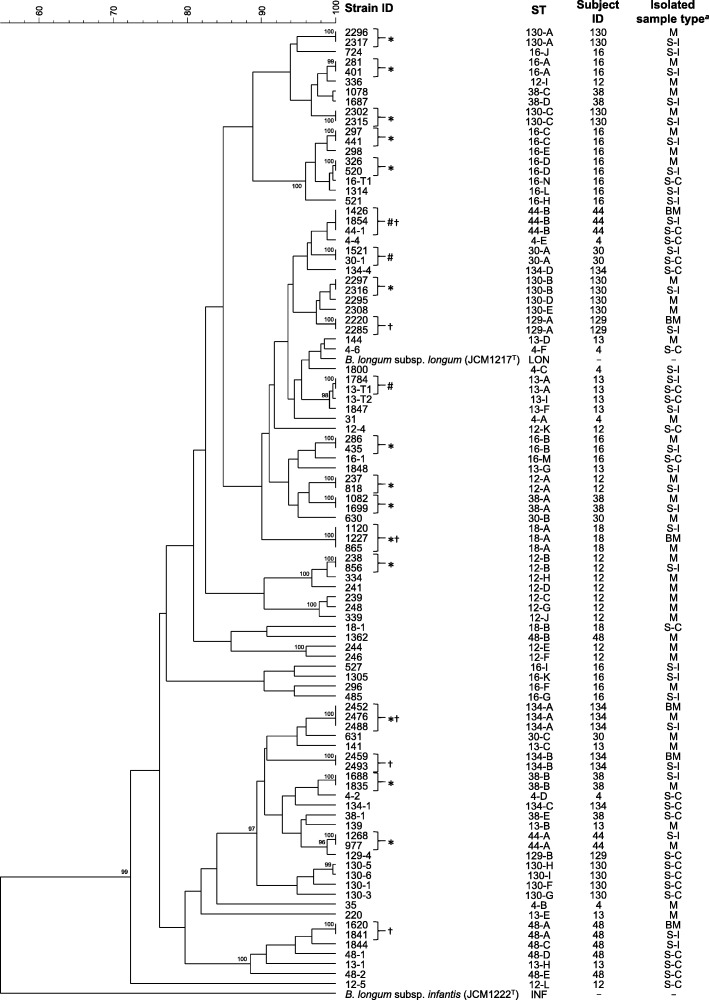
UPGMA dendrogram based on the allelic profiles. The dendrogram was constructed based on 247 positions of allelic profiles of representative strains, as well as the type strains of *B. longum* subsp. *infantis* and *B. longum* subsp. *longum*. *B. longum* subsp. *infantis* JCM 1222^T^ was used as the out-group. The scale bar shows the identical rate of allelic profile. Bootstrap values (%) based on 1000 replicates are given for nodes replicated at more than 95%. ^*a*^S-I, subject’s fecal sample collected in early infancy; S-C, subject’s fecal sample collected in childhood; M, mother’s prenatal fecal sample; BM, postnatal breast milk sample. *Monophyletic strain pair detected from both subjects’ fecal sample(s) in early infancy and their mothers’ prenatal fecal sample(s). ^†^Monophyletic strain pair detected from both subjects’ fecal sample(s) in early infancy and their mothers’ postnatal breast milk sample(s). ^#^Monophyletic strain pair detected from subjects’ fecal samples in both early infancy and childhood

For the detailed comparison of the nucleotide sequences in the MLST loci, further clustering analysis was conducted. Among the strains sharing the same ST, we selected 96 representative strains detected at the earliest sampling points of the subjects’ fecal samples collected in early infancy and childhood, as well as from their respective mothers’ perinatal samples (Additional file 1: Table S3). The results from cluster analysis based on the 247 positions of allelic profiles (Additional file 1: Table S4) indicated that there were no distinct clusters composed of the long-term colonizers or the strains shared between subjects’ fecal samples and their mothers’ perinatal samples (Fig. 2). Although some clusters composed of the strains originating from a specific subject or sample type were suggested, the general composition of the dendrogram did not reflect the source subject or the isolated sample type of the strains.

Upon comparison of the allelic profiles between the strains detected from subjects’ fecal samples in childhood and those of their closely related strain, 13-T2 (ST 13-I) and 16-T1 (ST 16-N) showed highly similar profiles (99.6% identity with one divergent position) compared to those of their relatives detected from the same subjects’ fecal samples in early infancy, 1784 (ST 13-A) and 520 (ST 16-D), respectively (Fig. 2).

### Amplified fragment length polymorphism profiles of long-term colonizers

**Fig. 3:**
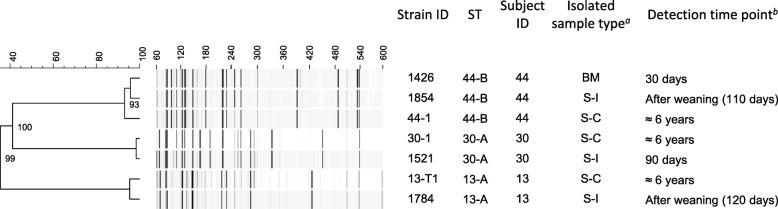
AFLP profiles of the representative strains belonging to three monophyletic strains classified as long-term colonizers. The UPGMA dendrogram was constructed based on an average of 238 ± 14 fragments of the AFLP profile. Cophenetic correlation is given at each node. The scale bars for similarity score (%) and fragment length (bp) are shown above the dendrogram and AFLP profile image, respectively. ^*a*^ S-I, subject’s fecal sample collected in early infancy; S-C, subject’s fecal sample collected in childhood; BM, postnatal breast milk sample. ^*b*^Calculated from the delivery date

In order to investigate the genomic similarity among three monophyletic strains (ST 13-A, 30-A, and 44-B) classified as long-term colonizers (Fig. 1), amplified fragment length polymorphism (AFLP) analysis was conducted. An average of 238 ± 14 fragments was detected from each of the seven representative strains. Based on the AFLP profile, the representative strains belonging to each ST were classified into distinct clusters with cophenetic correlation coefficients of > 99% (Fig. 3). In these clusters, highly similar AFLP profiles were observed among the strains (95.4, 98.4%, and 93.1–95.8% for ST 13-A, 30-A, and 44-B, respectively), suggesting that considerable genomic similarity was conserved within each set of monophyletic strains.

### Composition of major fecal *Bifidobacterium*

**Fig. 4:**
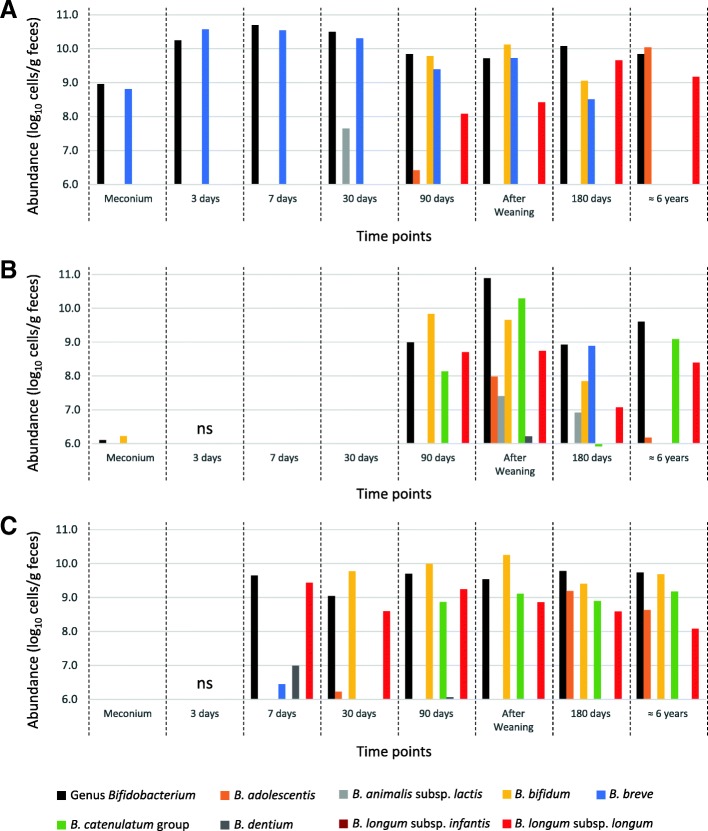
Compositions of major fecal *Bifidobacterium* of the subjects colonized by long-term colonizers. The fecal abundances of the genus *Bifidobacterium* along with seven species and three subspecies are shown for three subjects, (**a**) Subject 13, (**b**) Subject 30, and (**c**) Subject 44. ns: no sample was collected

In order to trace the population dynamics in the genus *Bifidobacterium,* including the long-term colonizers, the abundance of eight species and subspecies of the major fecal *Bifidobacterium* was measured by quantitative polymerase chain reaction (qPCR) for the subjects’ fecal samples collected in early infancy and childhood (Additional file 1: Table S5). The species and subspecies targeted in this analysis comprise most of the population of the genus *Bifidobacterium* at each time point, regardless of the subjects. Among the three subjects from whom monophyletic strains were detected in both early infancy and childhood (Subject IDs: 13, 30, and 44), *B. longum* subsp. *longum* was detected in early infancy from 90, 90, and 7 days of age, respectively, with the abundance of each strain being 8.08–9.65, 7.07–8.74, and 8.59–9.43 log_10_ cells/g feces, respectively (Fig. 4 and Additional file 1: Table S5). Except for subject 13 at 180 days of age and subject 44 at 7 days of age, *B. longum* subsp. *longum* was not the most dominant component of *Bifidobacterium,* but rather co-existed with other predominant and dominant species (i.e., *Bifidobacterium adolescentis, Bifidobacterium bifidum*, *Bifidobacterium breve*, and *Bifidobacterium catenulatum* group).

## Discussion

### Intra-species diversity in *B. longum* subsp. *longum*

From 12 out of the 49 subjects, *B. longum* subsp. *longum* isolates were obtained from the fecal samples collected in both early infancy and childhood. Several strains were observed in some subjects’ fecal samples collected in early infancy and childhood, as well as in the prenatal fecal samples of their mothers (Fig. 1 and Table 1). Although extensive intra-species bacterial diversity has been reported in the infant gut microbiota [11, 24], our result showed that the intra-species diversities of *B. longum* subsp. *longum* in the human gut exist not only in the infant but also in the child as well as in the prenatal mothers. Ellegaard and Engel have demonstrated some mechanisms hypothesized to facilitate strain co-existence (i.e., microniche differentiation, host selection phage selection, and cross-feeding/metabolic interdependency) [25]. In addition, Odamaki et al. showed that *B. longum* subsp. *longum* strains are commonly transmitted among family members [26]. Together, these finding imply that several strains of *B. longum* subsp. *longum* that have been transmitted among the family members could coexist in the gut as the result of different selection pressures. Consistent with these findings, in the current study, strains with the same ST were not found to be shared among different subjects’ fecal samples and their mothers’ perinatal samples despite the intra-species diversities (Fig. 1).

### Long-term colonization of *B. longum* subsp. *longum*

Results from our current study revealed that three monophyletic strains (ST 13-A, 30-A, and 44-B) persisted to colonize in the gut of individual subjects (Subject IDs: 13, 30, and 44) from early infancy for more than six years (Fig. 1). The close relation among the monophyletic strains was also supported by the results from the AFLP analysis (Fig. 3). Despite a high genomic similarity among each monophyletic stain, identical AFLP profiles were not observed among each of the strain combinations, suggesting the development of mutations during the colonization period in the human gut. This is consist with the previous report by Shkoporov et al. [21]. In addition to these long-term colonizers, the close relationship based on allelic profiles of two monophyletic strains detected from subjects 13 and 16 during childhood (ST 13-I and 16-N, respectively) implied that these strains evolved from common ancestors of the strains detected from the sample in early infancy of the same subjects (ST 13-A and 16-D, respectively) (Fig. 2).

In the present study, the long-term colonizers (ST 13-A, 30-A, and 44-B) were not detected before 90 days of age, although 23 out of the 31 monophyletic strains isolated in early infancy were detected earlier than this age (Fig. 1). Additionally, in subject 44, the initial major component of *B. longum* subsp. *longum,* ST 44-A, was thought to be replaced by ST 44-B between 7 and 110 days of age. These results implied that earlier colonization by strains was not likely to contribute to the long-term colonization. However, it is difficult to explain long-term colonization based only upon colonization timing. For example, 14 monophyletic strains that were suggested to be vertically transmitted from mothers’ gut were included in the “early” colonizers (Fig. 1). Considering that these strains were able to colonize in the mother’s gut as a major component of *B. longum* subsp. *longum*, it had been supposed that these strains should also be capable of adapting to the subject’s gut during childhood, which is more similar to the adult gut compared to that of the gut in early infancy. In addition to the characteristics of bacterial strain (e.g. colonization timing, adhesion factor on the human gut epithelium, and nutrient utilization), numerous other external factors should be considered that may affect the long-term colonization (e.g., host genetic background, immunological property, dietary habit, and antibiotic usage, as well as competition with other gut microbiota). Recent studies suggested that among numerous factors, host diet has the greatest impact on microbial colonization in human gut [27]. A detailed analysis considering host diet and nutrient utilization properties for our long-term colonizers may provide information to explain the long-term colonization of this species in human gut. Owing to the limited the number of subjects and long-term colonizers in this study, we did not investigate the association of these factors with long-term colonization in order to avoid reaching a biased conclusion. Larger numbers of subjects and more detailed follow-up of subject backgrounds will be required to answer this question. Further analysis on the long-term colonizers, together with those reported in previous studies (e.g. genome comparison) [20, 21], would also likely contribute to our understanding of factors underlying the long-term colonization of this species in the human gut from the perspective of bacterial strain characteristics.

Several studies have reported an association between the gut microbial composition during early infancy and the risk of disease in the following life stages, including allergies, asthma, and obesity [6–8]. Our results suggested long-term colonization of bacterial strains from early infancy and implied that a bacterial strain colonized in the human gut during this period might have a longitudinal effect on the host health. Currently, some strains belonging to the genus *Bifidobacterium* (e.g. *Bifidobacterium animalis* subsp. *lactis*, *B. breve,* and *B. longum* subsp. *infantis*) have been proposed for use as probiotics targeting infants [28]. Our findings also showed the possibility that probiotics targeting infants might have longitudinal effects.

### Origin of long-term colonizers

Although long-term colonizers were detected as the major component of *B. longum* subsp. *longum*, the source of these remained unclear. The cluster analysis based on the allelic profiles showed no phylogenetic relationship between long-term colonizers (Fig. 2), suggesting that the characteristics were not shared within a specific lineage of the strains belonging to this species. Although our study showed that one of the monophyletic strains (ST 44-B) was detected from breast milk (at 30 days after delivery) before the strain was detected from the subject fecal sample in early infancy (Fig. 1), we are unable to specify the precise origin of the strain. Possible origins of long-term colonizers would likely be family members of the subjects and the surrounding environment. For example, recent studies have shown a highly frequency of transmission of *B. longum* subsp. *longum* strains, not only from mother-to-infant, but also between other family members [26], as well as the existence of a distinct microbiome in each family’s home [29].

### Persistence of long-term colonizers among other predominant *Bifidobacterium* species

The qPCR results showed that *B. longum* subsp. *longum* continued to be a dominant component of fecal *Bifidobacterium* (7.07 to 9.65 log_10_ cells/g feces) once this species colonized in the subject gut (Fig. 4). Since strain-specific quantification was not conducted in this study, we were unable to trace the actual dynamics for the abundance of long-term colonizers. Since only strains belonging to ST 30-A and 44-B were detected from the subjects’ fecal samples after their colonization (Fig. 1), it was suggested that a considerable abundance of these strains persisted to colonize in the subject’s gut for more than six years. In subject 44, the initial major component of *B. longum* subsp. *longum* (ST 44-A) was thought to be replaced by a long-term colonizer (ST 44-B) between 7 and 110 days of age. However, the precise timing of the replacement was unclear since no strain was detected at 30 days of age owing to limitations of the culturing approach. Moreover, for subject 13, because several strains were detected at 180 days and six years of age, the exact abundance of the long-term colonizer (ST 13-A) was not estimated at these time points.

Our qPCR results also revealed that the long-term colonizers were able to co-exist with other predominant species of *Bifidobacterium*. As previous studies have demonstrated the divergence of nutrient utilization (e.g. food-derived or human milk oligosaccharides and host-produced glycans) in *Bifidobacterium* at the species or strain level [30–32], this implied that segregation of nutrient utilization may constitute one of the factors that might enable a long-term colonizer to persist among other predominant species of *Bifidobacterium*.

## Conclusions

Our results revealed the existence of monophyletic strains belonging to *B. longum* subsp. *longum* that colonized in the same subject’s gut from their early infancy for more than six years, confirming the existence of long-term colonizers from this period. Our data also suggested the co-existence of these long-term colonizers with other predominant species of *Bifidobacterium*. These findings may help to understand how this bacterial species evolved as a symbiont in the human gut and also may indicate the possibility of longitudinal effects of the strains belonging to this bacterial species introduced in the human gut during early infancy. In addition, the bacterial resources generated in this study will contribute to future studies aimed at clarifying the mechanisms of bacterial long-term colonization in the human gut. Our findings also suggested the importance of a microbial-strain colonizing in early infancy relative to their succession and showed the possibility that probiotics targeting infants might have longitudinal effects.

## Methods

### Fecal sample collection

The current follow-up study was conducted in the area of Antwerp (Belgium) in 2016 (ISRCTN25216339). We recruited 49 subjects who had previously completed all the procedures of our previous study (ISRCTN66704989). The fecal samples used in this study were collected after at least five days of a washout period during which subjects were prohibited from taking fermented milk products. Following the methods of our previous studies [13, 17], a portion of freshly voided feces was collected in a sterile glass tube containing 6 ml of anaerobic transport medium for the cultivation of *Bifidobacterium* and in a sterile tube containing 2 ml RNAlater® (Thermo Fisher Scientific, Waltham, MA, USA) for qPCR analysis. Samples were kept at 4 °C after the collection and sent to the laboratory within one day after defecation. After arrival to the laboratory, the fecal sample for qPCR was washed twice with 1 ml phosphate buffered saline and stored at − 20 °C until DNA extraction.

### Bifidobacterial isolation and taxonomic identification

In the follow-up study, bifidobacteria were isolated from fecal samples and their DNAs were extracted, also according to Makino et al. [13] with slight modifications. Briefly, serial dilutions of homogenized fecal samples were prepared with saline and inoculated onto a selective medium for *Bifidobacterium* (TOS propionate agar; Merck Co. Ltd., Darmstadt, Germany) supplemented with 50 μg/ml mupirocin (TOS-M agar), or TOS-M agar containing 16 μg/ml tetracycline (TOS-MT agar). Isolates obtained from TOS-MT agar were indicated with a “T” at the end of the strain ID. After anaerobic culturing at 37 °C for 72 h, two to three colonies showing different colony morphologies were isolated. Additional single-colony isolation was carried out at least two times by using the same agar plate.

For DNA extraction, the purified bacterial isolates were anaerobically cultured in GAM medium (Nissui Co., Tokyo, Japan) supplemented with 1% glucose at 37 °C for 24 h. Cellular DNA was extracted by means of phenol/glass bead extraction as previously described [33] and used for subsequent taxonomic identifications. Initially, the species was determined based on partial nucleotide sequence of the 16S rRNA gene. The whole 16S rRNA gene was amplified by using the universal forward primer for *Bifidobacterium* BI8 and the universal reverse primer 15R as previously described [34]. After purifying the amplicon using the Ampure® XP Kit (Beckman-Coulter, Brea, CA, USA), the nucleotide sequence of the target region was determined using the primers BI8 and 520R by using BigDye Terminator v3.1 chemistry (Life Technologies, Carlsbad, CA, USA) on a 3130xl Genetic Analyzer (Life Technologies). The determined sequence was searched against NCBI BLAST (https://blast.ncbi.nlm.nih.gov/Blast.cgi) and the species was determined based on the highest score. If the bacterial isolate was identified as *B. longum*, the subspecies-specific PCR was conducted by using specific primers for *Bifidobacterium longum* subsp. *infantis* (BiINF-1 and BiINF-2) and those for *B. longum* subsp. *longum* (BiLON-1 and BiLON-2), as previously described [35]. The reaction mixture (25 μl) contained 10 mM Tris-HCl (pH 8.9), 50 mM KCl, 1.5 mM MgCl_2_, 200 mM each dNTP, 0.5 U Taq DNA polymerase (TaKaRa, Shiga, Japan), 0.4 mM of each respective primer, and 10 ng DNA template. The PCR amplification program was the same as that previously described [35] with decreased PCR cycles (i.e. 30 cycles) and an extended extension step (i.e. 50 s). Specific primer sequences for PCR are listed in Table 2.

**Table 2:** Primers used in this study

Primer Name	Target	Sequence (5′ - 3′)	Reference
Amplification and sequencing of 16S rRNA gene for *Bifidobacterium*			
BI8	16S rRNA gene	GGGTTYCGATTCTGGCTCAGGATG	[34]
15R		AAGGAGGTGATCCARCCGCA	
520R		ACCGCGGCTGCTGGC	
Subspecies-specific PCR for *Bifidobacterium longum*			
BiINF-1	*Bifidobacterium longum* subsp. *infantis*	TTCCAGTTGATCGCATGGTC	[35]
BiINF-2		GGAAACCCCATCTCTGGGAT	
BiLON-1	*Bifidobacterium longum* subsp. *longum*	TTCCAGTTGATCGCATGGTC	[35]
BiLON-2		GGGAAGCCGTATCTCTACG	
MLST for *Bifidobacterium longum* subsp. *longum* strains			
Blon-clpC-F	*clpC*	CCTGAAGAAGGTGCTGAAGG	[22]
Blon-clpC-R		TTCTCCTGCTTGTCGCGCAGT	
Blon-dnaG-F	*dnaG*	GTTGCCGTAGATTTGGGCTTGG	[22]
Blon-dnaG-R		ATGACTTCGGTGTTCCGCAC	
Blon-dnaJ-F	*dnaJ*	GCTGAGCAAGAAGGAAGATCGC	[22]
Blon-dnaJ-R		TGAACTTCTTGCCGTCCACGG	
Blon-fusA-F	*fusA*	CACCATCAAGGAGAAGCTGG	[22]
Blon-fusA-R		ACGAGCTTGCCGTAGAACG	
Blon-gyrB-if1	*gyrB*	AAGTGCGCCGTCAGGGCTT	[22]
Blon-gyrB-R		GTGTTCGCGAAGGTGTGCAC	
Blon-purF-F2	*purF*	CGGCTGAACTCGAAGAC	This study
Blon-purF-R2		GTTGAGCGCTTCCTTGAG	
Blon-rpoB-F	*rpoB*	AGACCGACAGCTTCGATTGG	[22]
Blon-rpoB-R		AACACGATGGCGGACTGCTT	
AFLP analysis			
MseI adapter 1	Restriction site of MseI	TACTCAGGACTCAT	[22]
MseI adapter 2		GACGATGAGTCCTGAG	
MspI adapter 1	Restriction site of MspI	CTCGTAGACTGCGTACA	[22]
MspI adapter 2		CGTGTACGCAGTCTAC	
Preselective MseI	MseI adapter	GATGAGTCCTGAGTAA	[22]
Preselective MspI	MspI adapter	GACTGCGTACACGGA	[22]
Selective MseI-T	MseI adapter	GATGAGTCCTGAGTAAT	[22]
Selective MspI-A	MspI adapter	FAM^a^-GACTGCGTACACGGAA	[22]
Quantification of fecal *Bifidobacteirum*			
g-Bifid-F	Genus *Bifidobacterium*	CTCCTGGAAACGGGTGG	[17]
g-Bifid-R		GGTGTTCTTCCCGATATCTACA	
BiADOg-1a	*Bifidobacterium adolescentis* group^b^	CTCCAGTTGGATGCATGTC	[17]
BiADOg-1b		TCCAGTTGACCGCATGGT	
BiADO-2		CGAAGGCTTGCTCCCAGT	
Bflact2	*Bifidobacterium animalis* subsp. *lactis*	GTGGAGACACGGTTTCCC	[17]
Bflact5		CACACCACACAATCCAATAC	
BiBIF-1	*Bifidobacterium bifidum*	CCACATGATCGCATGTGATTG	[17]
BiBIF-2		CCGAAGGCTTGCTCCCAAA	
BiBRE-1	*Bifidobacterium breve*	CCGGATGCTCCATCACAC	[17]
BiBRE-2		ACAAAGTGCCTTGCTCCCT	
BiCATg-1	*Bifidobacterium catenulatum* group^c^	CGGATGCTCCGACTCCT	[17]
BiCATg-2		CGAAGGCTTGCTCCCGAT	
BiDEN-1	*Bifidobacterium dentium*	ATCCCGGGGGTTCGCCT	[17]
BiDEN-2		GAAGGGCTTGCTCCCGA	
BiINF-1	*Bifidobacterium longum* subsp. *infantis*	TTCCAGTTGATCGCATGGTC	[17]
BiINF-2		GGAAACCCCATCTCTGGGAT	
BiLON-1	*Bifidobacterium longum* subsp. *longum*	TTCCAGTTGATCGCATGGTC	[17]
BiLON-2		GGGAAGCCGTATCTCTACG	

^a^6-carboxyfluorescein

^b^The *B. adolescentis* group includes *B. adolescentis* genotypes A and B

^c^The *B. catenulatum* group includes *B. catenulatum* and *Bifidobacteium pseudocatenulatum*

### Multilocus sequencing typing analysis

In order to remove duplicate isolates obtained from the same sample and to investigate the identity among the strains obtained from different samples, the bifidobacterial isolates were distinguished at strain level by MLST analysis, as previously described [22], based on the nucleotide sequences of seven housekeeping genes, i.e. *clpC* (class III stress response-related ATPase with chaperone activity), *dnaG* (DNA primase), *dnaJ* (chaperone protein DnaJ), *fusA* (GTP-binding protein chain elongation factor G), *gyrB* (the β subunit of DNA gyrase), *purF* (amidophosphoribosyltransferase) and *rpoB* (the β subunit of RNA polymerase).

In our previous studies [13, 22, 23], bifidobacterial isolation was carried out from the subjects’ fecal samples collected at seven time points of early infancy: meconium, 3, 7, 30, 90, and 180 days of age, as well as after weaning (one week after the introduction of solids; at 140 ± 20 days for the subjects recruited in the follow-up study). In this study, we focused on the 12 subjects from whose fecal samples *B. longum* subsp. *longum* isolates were obtained in both early infancy and childhood (i.e., at the follow-up study conducted at approximately six years of age) (Additional file 1: Table S1). We investigated the existence of long-term colonizers (i.e., strains persisting to exist in the same subject’s gut from early infancy to childhood), as well as whether such strain(s) were transmitted from the mother’s prenatal gut to the subject’s gut or shared between the mother’s postnatal breast milk and the subject’s gut. Therefore, we confirmed the identities of the isolates obtained from the subjects’ fecal samples collected in early infancy and childhood as well as from the mothers’ perinatal samples (i.e., prenatal fecal sample collected twice with at least one-week interval before delivery and postnatal breast milk samples collected at 7 and 30 days after delivery) (Additional file 1: Tables S1 and S2).

The amplification of target genes for MLST analysis was conducted in 25 μl of reaction mixture containing 10 mM Tris-HCl (pH 8.9), 50 mM KCl, 1.5 mM MgCl_2_, 200 mM each dNTP, 0.5 U Taq DNA polymerase (TaKaRa), 0.4 mM of each respective primer (Table 2), and 10 ng DNA template. The PCR amplification program consisted of an initial heating step at 94 °C for 5 min; 30 cycles of 94 °C for 30 s, 57 °C for 30 s, and 72 °C for 1 min; and a final extension step at 72 °C for 10 min. The procedures for amplicon purification and sequencing were the same as those for species identification except for the primers used in sequencing, which corresponded to those used for the amplification of the target genes. In addition to the sequences determined in this study and our previous study [13], we extracted the corresponding housekeeping gene sequences from the genome sequences of the type strains of *B. longum* subsp. *infantis* (JCM 1222^T^) and *B. longum* subsp. *longum* (JCM 1217^T^) [4] and used them in the following analysis.

The sequences were aligned for each gene based on the MUSCLE algorism v3.8.1 [36] mounted on GENETYX® Ver.12.0.5 (GENETYX, Tokyo, Japan). For each isolate, in total 2902 bp (*clpC*, 479 bp; *dnaG*, 305 bp; *dnaJ*, 297 bp; *fusA*, 498 bp; *gyrB*, 396 bp; *purF*, 431 bp; *rpoB*, 496 bp) of nucleotide sequences were imported into BioNumerics® version 7.6 (Applied-Maths, Sint-Martens-Latem, Belgium). Each distinct gene sequence was assigned to an allelic number, and each unique combination of seven allelic numbers was assigned to an ST. The isolates showing the distinct ST for each sample at each sampling point were classified as the same strain.

### Clustering analysis based on allelic profiles

Clustering analysis was also carried out using BioNumerics® version 7.6. Together with the 96 representative strains (Additional file 1: Table S3), the type strains of *B. longum* subsp. *infantis* (JCM 1222^T^) and *B. longum* subsp. *longum* (JCM 1217^T^) were also included in this analysis. Among the 2902 bp of the aligned nucleotide sequences of the seven housekeeping genes, 247 positions of the allelic profile were detected as those at which identical sequence was not shared among all of the strains used for this analysis (Additional file 1: Table S4). The categorical coefficient was calculated based on the allelic profiles and the dendrogram was constructed on the basis of the unweighted pair group method with arithmetic means (UPGMA) algorithm. The statistical reliability of the trees was evaluated by bootstrap analysis of 1000 replicates [37] and the nodes replicated at more than 95% were regarded as statistically reliable.

### Amplified fragment length polymorphism analysis

AFLP analysis was conducted following the methodology described previously [22] with slight modifications. Seven representative strains belonging to ST 13-A, 30-A, and 44-B (Additional file 1: Table S3) were subjected to AFLP analysis. Five microliter of restriction reaction mixtures contained 1 × CutSmart® Buffer (New England BioLabs, Ipswich, MA), 5 U MseI (New England BioLabs), 5 U MspI (New England BioLabs) and 30 ng of DNA template. The restriction reaction was conducted at 37 °C for 2 h. Prior to ligation, equal amounts of adapters for MseI and MspI (Table 2) were separately mixed, denatured at 95 °C for 5 min, and left at room temperature for 5 min. Ligation was conducted in 10 μl of reaction mixtures containing 1 × T4 DNA ligase buffer (New England BioLabs), 2 μM MseI adapters, 2 μM MspI adapters, 40 U T4 DNA ligase (New England BioLabs), and 5 μl of digested DNA, with incubating at 20 °C for 2 h. The digested and ligated DNA was diluted 10-fold in Tris-EDTA buffer and used as template for the preselective PCR.

Preselective PCR was conducted in 10 μl of reaction mixture containing 10 mM Tris-HCl (pH 8.9), 50 mM KCl, 3 mM MgCl_2_, 250 μM each dNTP, 0.25 U *Taq* DNA polymerase (TaKaRa), 2.5 μM of each preselective primer (Table 2), and 1 μl of template DNA. The PCR amplification program was the same as that previously described [22]. The amplicon was diluted 100-fold in Tris-EDTA buffer and used as template for the selective PCR.

Selective PCR was conducted in 10 μl of reaction mixture containing 10 mM Tris-HCl (pH 8.9), 50 mM KCl, 3 mM MgCl_2_, 100 μM each dNTP, 0.25 U *Taq* DNA polymerase (TaKaRa), 30 nM of each selective primer (Table 2), and 1 μl of diluted amplicon of preselective PCR. The PCR amplification program was the same as that previously described [22]. Nine microliters of Hi-Di formamide (Life Technologies) and 1 μl of GeneScan™ 600 LIZ® size standards (Life Technologies) were mixed with 1 μl of the selective PCR products. Followed by denaturing at 95 °C for 1 min, the selective PCR products were detected using a 3130xl Genetic Analyzer (Life Technologies). Output in FSA format was imported into BioNumerics® version 7.6 (Applied-Maths). AFLP analysis was conducted for the fragments ranging from 60 to 600 bp and a threshold of 1% was used for position tolerance. A UPGMA dendrogram was constructed based on Pearson correlation coefficients. The quality of each branch was evaluated by calculating the cophenetic correlation.

### Fecal DNA extraction and quantification of major fecal *Bifidobacterium*

The composition of the major fecal *Bifidobacterium* were analyzed by qPCR for the subjects’ fecal samples collected in early infancy and childhood. According to our previous study [17], fecal DNA was extracted from freeze-stored phosphate buffered saline-suspended fecal sample as described above and the abundances of the genus *Bifidobacterium,* along with that of seven species and three subspecies of fecal *Bifidobacterium* (*B. adolescentis*, *B. animalis* subsp. *lactis*, *B. bifidum*, *B. breve*, *B. catenulatum* group, *Bifidobacterium dentium, B. longum* subsp. *infantis*, and *B. longum* subsp. *longum*) were measured using the primer sets listed in Table 2. PCR amplification and detection were performed with an ABI PRISM 7900HT Sequence Detection System and SDS software (version 2.4.1; Thermo Fisher Scientific, Waltham, MA, USA). 10 μl of the reaction mixture containing 10 mM Tris-HCl, pH 8.9; 50 mM KCl; 1.5 mM MgCl_2_; 200 μM of each dNTP; 1:75,000 dilution of SYBR Green I (Thermo Fisher Scientific); 0.5 U of Taq DNA polymerase Hot start version (Takara Bio); 0.25 μM of each of the specific primers; and 1 μl of 10-fold, 100-fold, or 1000-fold diluted template DNA. The amplification program consisted of one cycle at 94 °C for 5 min; 40 cycles at 94 °C for 20 s, 55 °C for 20 s, and 72 °C for 50 s; and finally one cycle at 94 °C for 15 s followed by the melting curve measurement using gradient heating increments of 0.2 °C/s from 60 °C to 95 °C. Fluorescent products were detected during the last step of each cycle. For the subjects’ fecal samples collected in early infancy, the abundance data measured in our previous study [17] was used in this study.

## Additional files

Additional file 1: **Table S1.** Detailed information of the subjects participating in the follow-up study. **Table S2.** Count of *B. longum* subsp. *longum* isolates obtained from the samples collected from the 12 subjects and their mothers. **Table S3.** Detailed information of the representative strains and the type strains of *B. longum* subsp. *infantis* and *B. longum* subsp. *longum.* **Table S4.** Allelic profiles for the representative strains and the type strains of *B. longum* subsp. *infantis* and *B. longum* subsp. *longum* **Table S5.** Raw data of qPCR analysis (XLSX 128 kb)

## Abbreviations

AFLP: Amplified fragment length polymorphism
*clpC*: Class III stress response-related ATPase with chaperone activity
DNA: Deoxyribonucleic acid
*dnaG*: DNA primase;
*dnaJ*: Chaperone protein DnaJ
*fusA*: GTP-binding protein chain elongation factor G
*gyrB*: The β subunit of DNA gyrase
MLST: Multilocus sequence typing
PCR: Polymerase chain reaction
*purF*: Amidophosphoribosyltransferase
qPCR: Quantitative polymerase chain reaction
*rpoB*: The β subunit of RNA polymerase
rRNA: Ribosomal ribonucleic acid
ST: Sequence typing
UPGMA: Unweighted pair group method with arithmetic means
